# Association between cMIND diet and hypertension among older adults in China: a nationwide survey

**DOI:** 10.1007/s40520-024-02842-3

**Published:** 2024-09-05

**Authors:** Yazhu Wang, Yu Zhang, Xinrong Zeng, Xiaobing Xian, Jingyu Chen, Tengfei Niu

**Affiliations:** 1 Department of Cardiology, Shapingba Hospital affiliated to Chongqing University (Shapingba District People’s Hospital of Chongqing), Chongqing, 400030 China; 2 School of Public Health, Chongqing Medical University, Chongqing, 400016 China; 3 The Thirteenth People’s Hospital of Chongqing, Chongqing, 400053 China; 4 Chongqing Geriatrics Hospital, Chongqing, 400053 China; 5 Department of Basic Courses, Chongqing Medical and Pharmaceutical College, Chongqing, 401331 China

**Keywords:** cMIND, Hypertension, CLHLS, Older adults

## Abstract

**Background:**

Existing research indicates that the Mediterranean diet has a positive impact on preventing and treating hypertension. However, its specific effect on hypertension among elderly Chinese individuals is unclear.

**Aims:**

The objective of this research was to explore the association between the Chinese version of the Mediterranean-DASH Intervention for Neurodegenerative Delay (cMIND) diet and hypertension among elderly Chinese individuals, aiming to offer novel strategies for alleviating the burden of hypertension in this demographic.

**Methods:**

In this study, we used cross-sectional data published in 2018 by the China Longitudinal Health and Longevity Survey (CLHLS) to develop a binary logistic regression model to investigate the correlation between cMIND diet and hypertension in a Chinese elderly population. Restricted cubic spline was used to test for linear associations, and further subgroup analyses were performed to test for interactions.

**Results:**

In total, 7,103 older adults were included in the study, with a prevalence of hypertension of 39.0%. When the cMIND diet score was used as a continuous variable, a significant protective effect against hypertension was present (OR = 0.955, 95% CI:0.923–0.988, *p* = 0.008); when used as a categorical variable, this protective effect was still present at higher levels (compared to lower levels) of the cMIND diet (OR = 0.869, 95% CI: 0.760–0.995, *p* = 0.042).

**Discussion:**

Although the Mediterranean diet has great potential to reduce the chance of hypertension, it should also consider the effect on the Chinese population. The results of this study provide new ways to reduce the disease burden of hypertension in Chinese older adults and improve quality of life in later life.

**Conclusion:**

The cMIND diet can considerably reduce the risk of hypertension among older adults in China.

**Supplementary Information:**

The online version contains supplementary material available at 10.1007/s40520-024-02842-3.

## Introduction

A global report on hypertension, published by WHO in 2023, states that over one billion people are at risk of hypertension and its associated complications [1]. Global Burden of Disease 2019 study shows that the average prevalence of cardiovascular disease was 77–80% among individuals aged 60 to 80 and more than 85% in people over 80 years old [2]. The results of a study showed that the age-standardized mortality rate (ASMR) and age-standardized disability-adjusted life years rate (ASDR) for hypertension-associated diseases across China in 2019 were 153.34 /100,000 and 2844.27/100,000 respectively, which were approximately 3.50 times higher than those in Japan and Korea [3]. Research studies at the national level have shown that hypertension currently affects half of the elderly population in China. Among those aged 80 or above, the prevalence of hypertension is nearly 90% [4]. Hypertension stands as a prevalent chronic illness among older adults in China, significantly contributing to cardiovascular disease, which remains the primary cause of mortality globally [5, 6]. Hypertension usually causes complications encompassing cardiovascular disease (CVD), stroke, and kidney dysfunction, resulting in a diminished quality of life and shorter life expectancy [7]. A study on the Global Burden of Disease Study 2017 found that a significant contribution to stroke-related deaths (69%), ischemic heart disease fatalities (54%), and chronic kidney disease mortalities (43%) was traced back to hypertension [8]. Extensive research has revealed a robust correlation between the development of hypertension and a variety of risk factors, such as population aging, family history, obesity, smoking, alcohol consumption, and stress [4, 9–13]. The Global Sustainable Development Goals (SDGs) plan released by the World Health Organization mentions a projected 33% reduction in non-communicable diseases, including hypertension, by 2030 [14]. Therefore, it is urgent to find protective factors that can effectively reduce the probability of developing hypertension. De Pergola G. et al. concluded that a healthy lifestyle represents a cornerstone approach to reducing hypertension, and dietary patterns are the most influential variable on blood pressure [15]. DASH (Dietary Approaches to Stop Hypertension) dietary pattern is effective in controlling blood pressure levels in many studies and has substantial advantages in the management of hypertension [16–18]. In addition to the DASH diet, scholars have also widely noted the role of the Mediterranean diet in reducing hypertension.

The Mediterranean dietary pattern, stemming from the cultures bordering the Mediterranean Sea basin, represents a distinctive eating habit characterized by an abundance of fruits, vegetables, whole grains, legumes, nuts, and seeds, coupled with moderate consumption of dairy products, poultry, and fish while limiting the intake of red meat and sweets [19]. The Mediterranean diet emphasizes monounsaturated fats and polyphenols, which protect the heart and reduce atherosclerosis and cardiovascular diseases [20, 21]. A study showed that the Mediterranean diet is a healthful dietary model that minimizes the risk of non-communicable diseases [22]. Extensive research indicates that adopting the Mediterranean diet reduces the probability of developing hypertension [23–25]. However, due to the many differences in dietary patterns between China and the West, the existing Mediterranean dietary patterns are not entirely suitable for China with its diverse dietary culture [26]. In recent years, scholars have actively conducted research on the correlation between the Mediterranean diet and chronic illnesses in China and developed a MIND diet (including more whole grains and less refined flour or rice) suitable for the Chinese population (the Chinese version of the Mediterranean-DASH Intervention for Neurodegenerative Delay, cMIND) [27–32]. Existing studies have shown that in the Chinese elderly population, the Mediterranean diet significantly reduces the probability of developing chronic illnesses such as cognitive impairment (CI), age-related macular degeneration, and cardiovascular disease [27, 31, 33].

Nevertheless, the association between the Mediterranean diet and hypertension among Chinese older adults remains unknown. Further research evidence of its correlation is imperative to gain a comprehensive understanding of the influence of the Mediterranean dietary pattern on hypertension [15]. Hence, the objective of this research was to explore the association between the cMIND diet and hypertension among elderly Chinese individuals, aiming to offer novel strategies for alleviating the burden of hypertension in this demographic.

## Materials and methods

### Participants and process

The data utilized in this research was derived from the Chinese Longitudinal Healthy Longevity Survey (CLHLS). Launched in 1998, this project employs a multi-stage stratified sampling approach to identify participants, offering insights into the health conditions and well-being of elderly individuals aged 65 and above across 23 provinces in China. Data were collected one-on-one by trained professionals to ensure a representative and reliable sample. Subjects signed an informed consent form to agree to the survey, and the project was approved by the Biomedical Ethics Committee of Peking University, China (IRB00001052-13074).

**Fig. 1:**
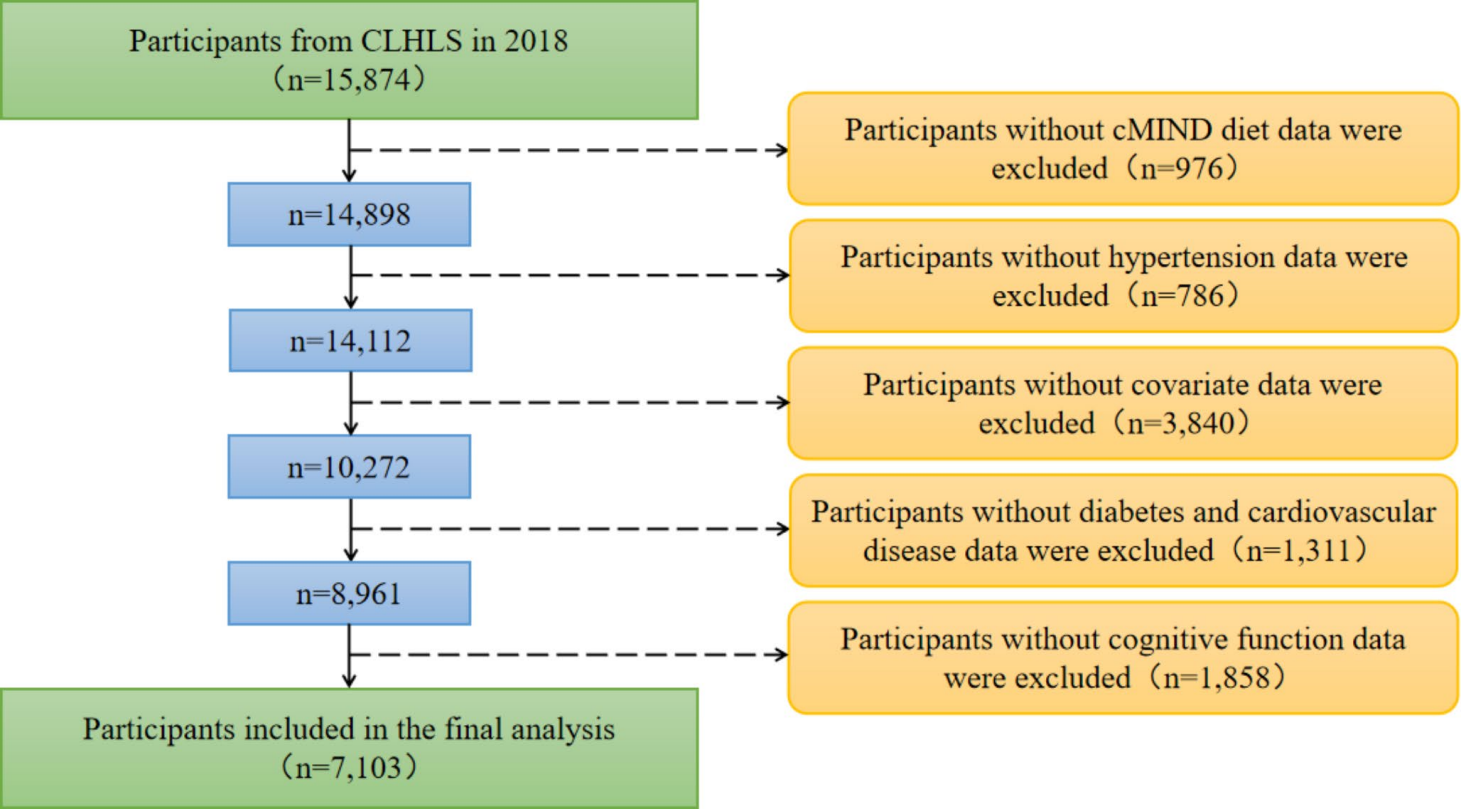
Flowchart of participant selection

In this study, we used cross-sectional data published in 2018 to explore the relationship between the cMIND diet and hypertension, and the inclusion criteria for the study population were (i) age ≥ 65 years and (ii) no missing data for covariates and critical variables. The data cleaning procedure is depicted in Fig. 1.

### Assessment of cMIND diet

Huang X et al. developed the cMIND diet scale for the Chinese population based on the MIND diet scale and CLHLS food frequency questionnaire and assessed its reliability in the population [29]. cMIND diet consists of 12 food items: fresh vegetables, mushrooms/algae, fresh fruits, fish, soya beans, nuts, garlic, tea, sugar/sweets, type of staple food, quantity of staple food, and cooking oil. Three items, staple food type, staple food quantity, and cooking oil, were scored as 0 or 1, and the remaining nine items were scored as 0, 0.5 or 1. The cMIND diet adherence was evaluated on a 0–12 scale, where a higher score signifies greater conformity to the diet. In this study, the cMIND diet scores were categorized into three groups according to quantiles: low (0–4), medium (4.5–5.5), and high (6–12), and assigned the values of 0, 1, and 2, respectively.

### Assessment of hypertension

This study designated hypertension as (1) a self-reported history of hospital-diagnosed hypertension; (2) systolic blood pressure ≥ 140 mmHg or diastolic blood pressure ≥ 90 mmHg. Two blood pressure measurements were taken during the survey, with an interval of at least 1 min between measurements, and the average of the two systolic and diastolic blood pressures was taken. A participant was classified as hypertension if either criterion was fulfilled [34, 35].

### Covariates

Based on the results of previous studies [31, 36, 37], we controlled for confounders in terms of basic characteristics, lifestyle, and chronic diseases of the study population to minimize the impact on the results, including gender (male or female), age (65–79 or ≥ 80), place of residence (urban or rural), living arrangement (living with family, living alone or in an institution), years of education (0, 1–6 and ≥ 7 years), economic status (bad, average or good), marital status (unmarried or married), BMI (< 18.5, 18.5–23.9, or > 23.9), and dichotomous variables (yes or no): smoking, alcohol, exercise, diabetes mellitus, cardiovascular disease, and CI. (As shown in Table S1 of the supplementary information)

CI was assessed on the MMSE scale of the CLHLS, which comprises 24 questions in five categories: general ability, reaction, attention and calculation, recall and language, and comprehension and self-coordination. In general ability, the question “In one minute, name the things that a person can eat” scored 7 points (< 7 counts as a raw score, ≥ 7 counts as 7 points), and in evaluating the remaining questions, a mark of 1 point was awarded for a precise response. In contrast, an inaccurate response or failure to complete the question resulted in 0 points. The cognitive function of older adults was evaluated on a scale ranging from 0 to 30, wherein a higher score indicated superior cognitive performance. A threshold of 24 points was established as a benchmark, and a score of more than 24 points was defined as cognitively normal, with a value of 0; a score equal to or less than 24 points was defined as cognitively impaired, with a value of 1 [38].

### Statistical analysis

Categorical variables were characterized by frequencies and percentages (n, %), while continuous variables that adhered to normal distribution were summarized with the mean and the standard deviation (M ± SD). χ2 tests and t-tests were introduced to compare disparities between participants with varying demographic attributes. We further developed three logistic regression models to analyze the correlation between cMIND diet (considering both continuous and categorical conditions) and hypertension. Model 1 did not control for any factors; model 2 controlled for sex, age, and BMI; and model 3 further controlled for place of residence, living arrangements, years of education, economic status, marital status, smoking, alcohol, exercise, diabetes, cardiovascular disease, and CI.

Employing RCS, we examined the presence of a nonlinear association between the cMIND diet and hypertension. In addition, we conducted subgroup analyses and interaction analyses by gender, age, place of residence, marital status, smoking, alcohol, exercise, diabetes, cardiovascular disease, and CI to test for the modified effects of these variables. Finally, sensitivity analyses demonstrated the robustness of the results. Statistical analyses were conducted using SPSS 26.0 and R 4.3.0, and a significance level of *p* < 0.05 was established.

## Results

### The characteristics of study participants

In total, 7,103 samples were incorporated in this research, of which the incidence of hypertension in the demographic was 39%. As shown in Table 1, the number of women (53.75%) was higher than that of men (46.25%), and the proportion of women suffering from hypertension (50.51%) was higher than that of men (49.49%). The older adults aged over 80 accounted for 57.81%, of which 58.27% were hypertension. The results of χ² analysis showed statistically significant differences between different cMIND diets, gender, living arrangement, economic status, smoking, alcohol, BMI, diabetes, cardiovascular disease, CI and prevalence of hypertension. The t-tests conducted on two independent samples revealed significant variations in cMIND diet scores among those with or without hypertension.

**Table 1:** Basic demographic characteristics of the participants

Variables	Hypertension, *n*(%)		7,103(100)	Statistic	*P*
	No 4,335(61.00)	Yes 2,768(39.00)			
cMIND, Mean ± SD	4.97 ± 1.67	4.76 ± 1.61	4.89 ± 1.65	t = 5.16	< 0.001
cMIND, *n*(%)				χ²=17.18	< 0.001
0–4	1,545 (35.64)	1,101 (39.78)	2,646 (37.25)		
4.5–5.5	1,500 (34.60)	953 (34.43)	2,453 (34.53)		
6–12	1,290 (29.76)	714 (25.79)	2,004 (28.21)		
Sex, *n*(%)				χ²=19.23	< 0.001
Male	1,915 (44.18)	1,370 (49.49)	3,285 (46.25)		
Female	2,420 (55.82)	1,398 (50.51)	3,818 (53.75)		
Age, *n*(%)				χ²=0.40	0.525
65–79	1,842 (42.49)	1,155 (41.73)	2,997 (42.19)		
≥80	2,493 (57.51)	1,613 (58.27)	4,106 (57.81)		
Residence, *n*(%)				χ²=0.69	0.408
Urban	2,450 (56.52)	1,592 (57.51)	4,042 (56.91)		
Rural	1,885 (43.48)	1,176 (42.49)	3,061 (43.09)		
Living arrangements, *n*(%)				χ²=11.64	0.003
With household member(s)	3,470 (80.05)	2,282 (82.44)	5,752 (80.98)		
Alone	735 (16.96)	435 (15.72)	1,170 (16.47)		
In an institution	130 (3.00)	51 (1.84)	181 (2.55)		
Education (years), *n*(%)				χ²=0.84	0.657
0	1,934 (44.61)	1,264 (45.66)	3,198 (45.02)		
1–6	1,550 (35.76)	964 (34.83)	2,514 (35.39)		
≥7	851 (19.63)	540 (19.51)	1,391 (19.58)		
Economic status, *n*(%)				χ²=10.48	0.005
Not wealthy	396 (9.13)	284 (10.26)	680 (9.57)		
General	3,016 (69.57)	1,977 (71.42)	4,993 (70.29)		
Wealthy	923 (21.29)	507 (18.32)	1,430 (20.13)		
Marital status, *n*(%)				χ²=2.37	0.124
Unmarried	2,305 (53.17)	1,420 (51.30)	3,725 (52.44)		
Married	2,030 (46.83)	1,348 (48.70)	3,378 (47.56)		
Smoking, *n*(%)				χ²=22.46	< 0.001
No	3,685 (85.01)	2,234 (80.71)	5,919 (83.33)		
Yes	650 (14.99)	534 (19.29)	1,184 (16.67)		
Drinking, *n*(%)				χ²=5.05	0.025
No	3,663 (84.50)	2,283 (82.48)	5,946 (83.71)		
Yes	672 (15.50)	485 (17.52)	1,157 (16.29)		
Exercise, *n*(%)				χ²=3.12	0.077
No	2,739 (63.18)	1,806 (65.25)	4,545 (63.99)		
Yes	1,596 (36.82)	962 (34.75)	2,558 (36.01)		
BMI, *n*(%)				χ²=236.19	< 0.001
<18.5	510 (11.76)	566 (20.45)	1,076 (15.15)		
18.5–23.9	2,079 (47.96)	1,539 (55.60)	3,618 (50.94)		
>23.9	1,746 (40.28)	663 (23.95)	2,409 (33.92)		
Diabetes, *n*(%)				χ²=117.79	< 0.001
No	3,779 (87.17)	2,630 (95.01)	6,409 (90.23)		
Yes	556 (12.83)	138 (4.99)	694 (9.77)		
Cardiovascular disease, *n*(%)				χ²=41.90	< 0.001
No	3,849 (88.79)	2,585 (93.39)	6,434 (90.58)		
Yes	486 (11.21)	183 (6.61)	669 (9.42)		
CI, *n*(%)				χ²=11.91	< 0.001
No	3,231 (74.53)	1,960 (70.81)	5,191 (73.08)		
Yes	1,104 (25.47)	808 (29.19)	1,912 (26.92)		

Notes: BMI—body-mass index; CI—Cognitive impairment; t—t-test; χ²—Chi-square test; SD—standard deviation

### Association between cMIND diet and hypertension

As depicted in Table 2, the logistic regression model found that when cMIND diet was used as a continuous variable, in model 1 without controlling for any variable, the risk of hypertension was reduced by 0.926 times for every one unit increase in the score of cMIND diet (OR = 0.926, 95% CI:0.899–0.954, *p* < 0.001); in models 2 and 3, which further controlled for covariates, the protective effect was reduced but still significant (model 2: OR = 0.945, 95% CI: 0.916–0.974, *p* < 0.001; model 3: OR = 0.955, 95% CI: 0.923–0.988, *p* = 0.008).

**Fig. 2:**
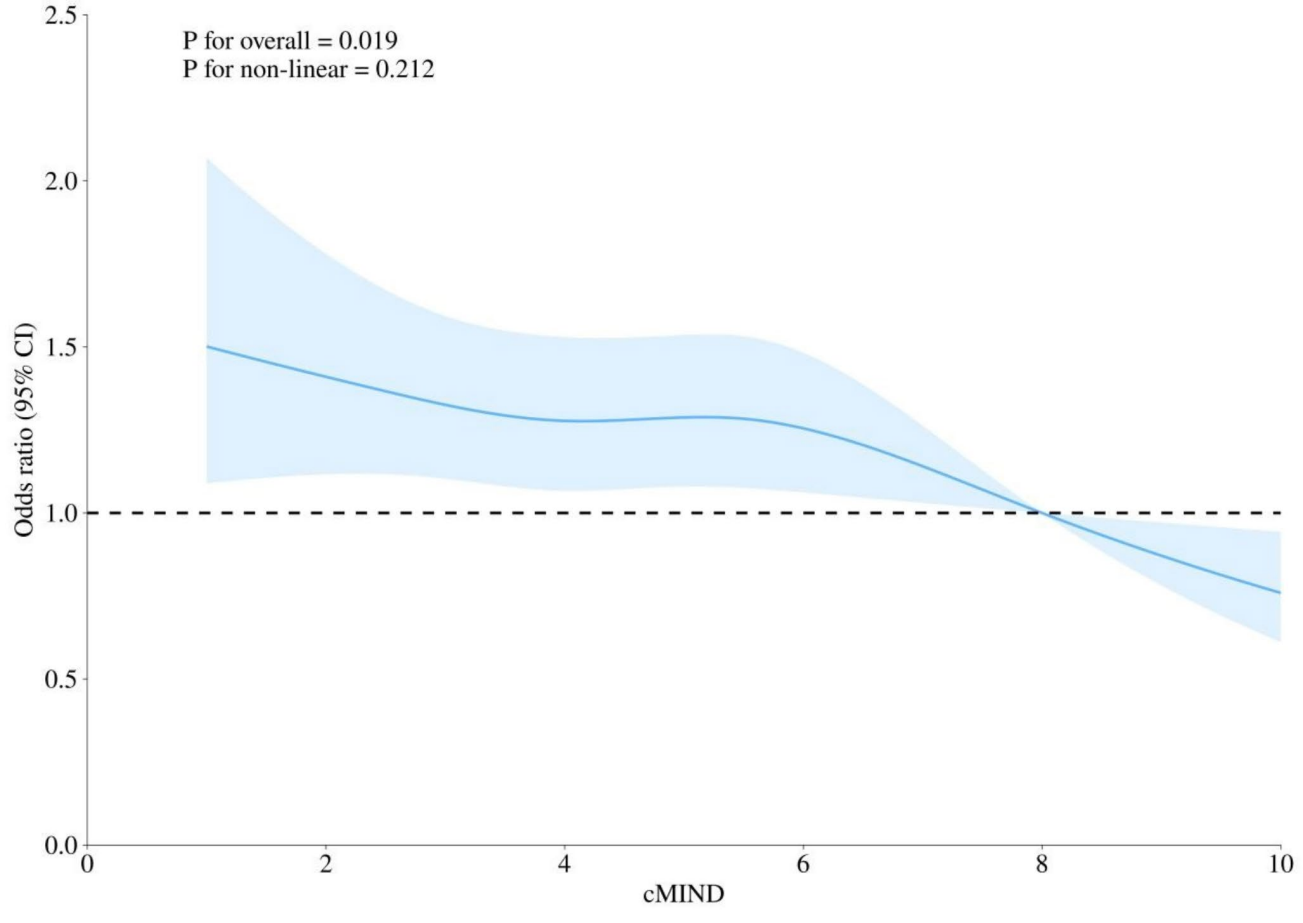
Restricted cubic spline for testing the hypothesis of non-linear correlation between cMIND diet and hypertension

When cMIND diet was used as a categorical variable, both medium and high levels of cMIND diet reduced the risk of hypertension in model 1 (medium level: OR = 0.892, 95% CI: 0.797 to 0.997, *p* = 0.045; high level: OR = 0.777, 95% CI: 0.689–0.875, *p* < 0.001); in model 2 and model 3, which further controlled for covariates, this protective effect persisted at higher levels of the cMIND diet (model 2: OR = 0.835, 95% CI: 0.736–0.947, *p* = 0.005; model 3: OR = 0.869, 95% CI: 0.760–0.995, *p* = 0.042). As shown in Fig. 2, the restricted cubic spline curve results demonstrated a statistically significant correlation between cMIND diet and hypertension in older adults in China (*p* < 0.05). Besides, the cMIND diet score revealed a linear trend with the prevalence of hypertension (p for non-linear = 0.212).

**Table 2:** Association of cMIND diet with hypertension among Chinese older adults

Variable	Model 1		Model 2		Model 3	
	OR(95%CI)	*P*	OR(95%CI)	*P*	OR(95%CI)	*P*
cMIND diet was used as a continuous variable	0.926 (0.899,0.954)	< 0.001	0.945 (0.916,0.974)	< 0.001	0.955 (0.923,0.988)	0.008
cMIND diet was used as a categorical variable (VS.Low)						
Medium	0.892 (0.797,0.997)	0.045	0.914 (0.815,1.026)	0.127	0.934 (0.831,1.051)	0.258
High	0.777 (0.689,0.875)	< 0.001	0.835 (0.736,0.947)	0.005	0.869 (0.760,0.995)	0.042

Notes: Mode1 without controlling for any variable; Model 2 controls for Sex, Age, BMI; Model 3 further controls for Residence, Living arrangements, Education, Economic status, Marital status, Smoking, Drinking, Exercise, Diabetes, Cardiovascular disease and CI

### Subgroup analysis

**Fig. 3:**
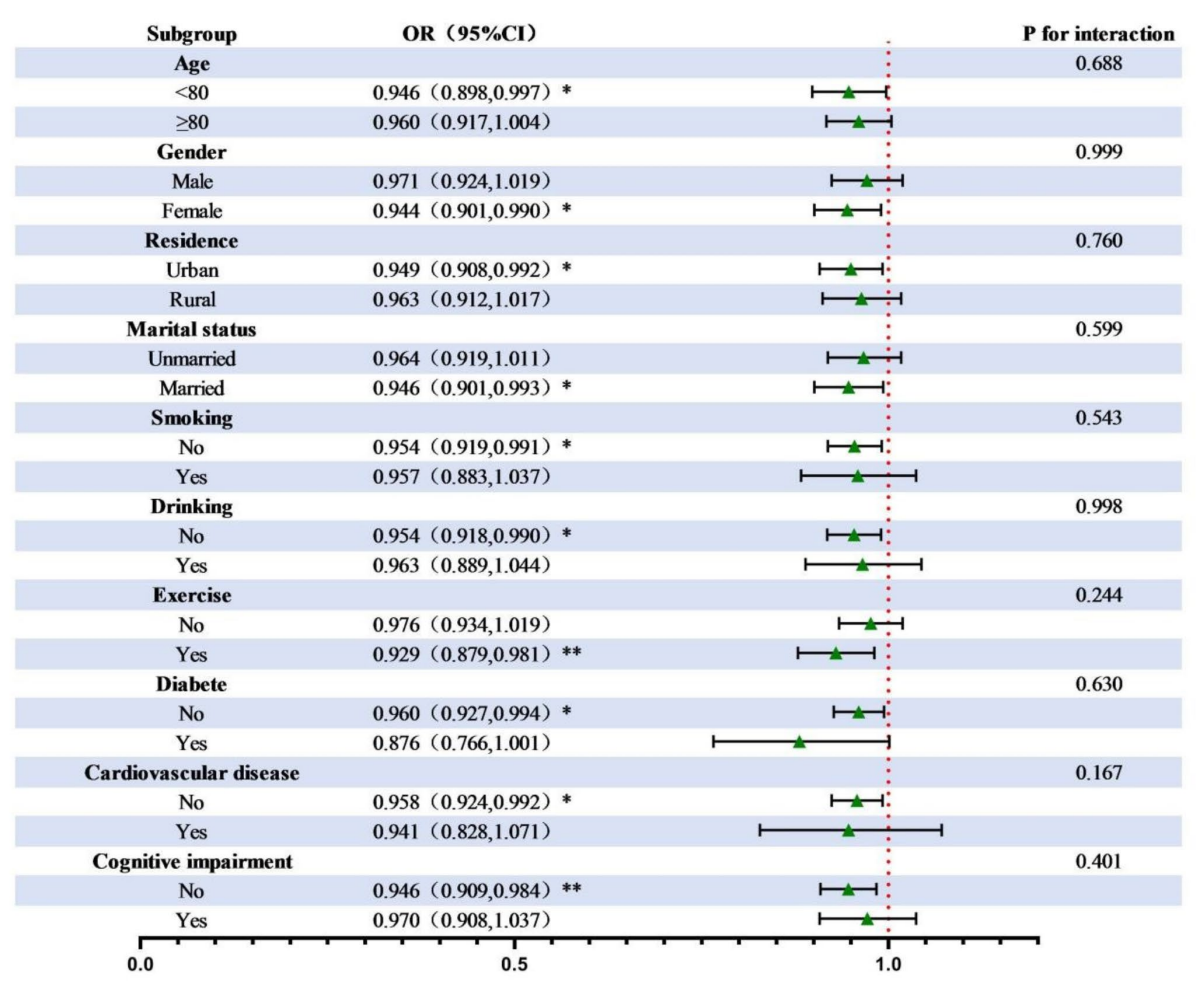
Associations of cMIND diet with hypertension among subpopulations. Notes: ** *p* < 0.01, * *p* < 0.05

Further subgroup analysis based on Model 3 covariates found that the correlation between the cMIND diet and the prevalence of hypertension was statistically significant in people < 80 years old, females, urban residents, the married, non-smokers, non-drinkers, exercisers, those without diabetes, those without cardiovascular disease, and those without CI. No significant interaction was found in the subgroup analysis, as shown in Fig. 3.

### Sensitivity analysis

As shown in Table S2 of the supplementary information, two sensitivity analyses were performed utilizing model 3 as the basis to ensure the robustness of the results: (i) older adults with diabetes and heart disease were excluded; (ii) older adults with CI (MMSE ≤ 24) were further excluded. The findings indicated that the protective effect of the cMIND diet on hypertension in older adults is still present regardless of whether the cMIND diet is a continuous or categorical variable.

## Discussion

Utilizing 2018 cross-sectional data from the CLHLS database, we found that the cMIND diet significantly reduced the probability of developing hypertension among older Chinese adults, and restricted cubic spline analysis showed a significant linear relationship. Further subgroup analysis found that the correlation between cMIND diet and hypertension was statistically notable in people < 80 years of age, females, urban residents, the married, non-smokers, non-drinkers, exercisers, those without diabetes, those without cardiovascular disease, and those without CI, and the interaction was nonexistent. The sensitivity analysis results further proved the robustness of the results.

The American Heart Association’s recommendations emphasize the pivotal role of dietary modifications in hypertension prevention and management, highlighting that adherence to healthy dietary patterns can drastically lower the risk of cardiovascular disease [39]. Scholars have long been interested in dietary approaches that can mitigate the probability of developing hypertension, with five significant dietary regimens exhibiting effectiveness in reducing such a condition: the DASH diet, the Mediterranean diet, the low-sodium diet, the vegetarian diet, and the combination diet [20]. Among them, the Mediterranean diet has received much attention for its great potential to reduce the chance of hypertension. A comprehensive assessment of substantial epidemiological data revealed that adherence to the Mediterranean diet leads to a marked decrease in blood pressure levels among patients with hypertension [23]; Song Y et al. concluded that the Mediterranean diet has practical value in both primary and secondary preventative measures against hypertension and that it is a new anti-hypertension dietary pattern [34]; Motamedi A et al. explored the chance of developing hypertension in Iranian adults and found that consistently observing the Mediterranean diet helps to prevent hypertension [24]. Apart from this, single foods in the typical Mediterranean dietary pattern have been confirmed by many studies to have a blood pressure-lowering effect, such as fresh vegetables, fruits, coarse grains, green tea, and other foods [40–43]. The dietary pattern used in this study is the Chinese version of the MIND diet based on traditional Mediterranean food by Hang X et al. It is widely reliable and representative of the Chinese population [29]. The cMIND diet, tailored to the dietary customs and geographic specifics of China, serves as an economical dietary model that fosters health and nutritional balance for the elderly population in the country [30]. According to the available studies, our study represents a novel exploration into the association between the cMIND diet and hypertension among elderly individuals in China.

The pathogenesis of hypertension is complex and varied and has long been a concern of scholars. As early as the 1950s, Irving Page proposed a complete theory of the pathogenesis of arterial hypertension, namely the Page mosaic theory [44]. As of today, there are two primary pathophysiological pathogenesis of hypertension - increased peripheral resistance and increased cardiac output. Elevated blood pressure in humans causes structural remodeling of blood vessels and rearrangement of vascular smooth muscle cells, resulting in reduced lumen diameter, hypertrophy of blood vessels, and increased vessel wall thickness [45]. In recent years, the treatment pattern of hypertension has changed from the initial pharmacological treatment to the present day’s equal emphasis on pharmacological treatment and lifestyle interventions, with a particular emphasis on nutritional changes. This study first concluded that the cMIND diet could significantly lower the chance of developing hypertension among Chinese older adults, suggesting that the cMIND dietary pattern could be considered an intervention to decrease the incidence of hypertension among them. Estruch R et al. suggested that the mechanisms of action of Mediterranean dietary interventions include promoting vasodilatation, improving endothelial function, decreasing fluid retention, and attenuating oxidative stress [46]. The Mediterranean diet emphasizes unsaturated fats and polyphenols, which can improve the function of the vascular endothelium and help control blood pressure [19]. For example, olive oil and fresh fruits and vegetables synergistically relax the blood vessels, thus lowering blood pressure [40, 47, 48]; monounsaturated fats and polyunsaturated fats found in nuts and fish have been associated with a reduction in blood pressure as well [49, 50]. Excessive sodium intake causes sodium retention, which in turn increases blood pressure [51, 52]. The pathogenesis of hypertension involves oxidative stress, which arises from an imbalance between the generation of reactive oxygen and nitrogen species and the antioxidant defense system, resulting in inflammation and subsequent vascular injury [53–57]. Fresh fruits and vegetables and plant sterols have been found to counteract oxidative stress and reduce vascular damage [58].

After stratifying participants by gender, age, place of residence, marital status, smoking, alcohol, exercise, diabetes, cardiovascular disease, and CI, the findings revealed that the correlation between cMIND diet and hypertension was statistically remarkable for people < 80 years of age, females, urban residents, the married, non-smokers, non-drinkers, exercisers, those without diabetes, those without cardiovascular disease, and those without CI. A study among married couples in India found that the prevalence of hypertension in males and females was 29.1% and 20.6% respectively. If the spouse suffered from hypertension, the study subjects were more likely to have hypertension [59]. In Nigeria, the chance of developing hypertension among those who are married is notably greater compared to individuals who are not married [60], which may be due to the influence of spouses’ similar eating habits and lifestyles [61]. Alcohol consumption and smoking often co-exist, and the average alcohol consumption of men is usually higher than that of women [12, 62]. Alcohol consumption can lead to vasoconstriction and increased heart rate, while smoking can produce oxidative stress and endothelial dysfunction, all of which significantly enhance the likelihood of hypertension [11, 63]. Furthermore, the association linking hypertension, dementia, and cognitive decline has been unequivocally established [64]. Studies have found that the MIND diet can improve language recognition, memory, and attention, and observing the MIND diet can also improve the occurrence of CI [65, 66].

Hypertension patients in China account for about 1/5 of the world’s patients, and there are three lows in the management and control of hypertension (low awareness rate, low treatment rate, and low control rate), which further increases the healthcare burden of cardiovascular diseases in China [67]. The Mediterranean diet has been widely used in China in recent years, but the opportunities for Chinese older adults to obtain and adhere to it are limited [68]. Given this, it may be necessary for various government departments and health practitioners to carry out health education to encourage older adults in China to adopt the cMIND dietary pattern.

## Conclusions

The study demonstrated that the cMIND diet significantly reduced the risk of hypertension among Chinese older adults, with a significant dose-response relationship. Further subgroup analyses demonstrated the variability of this association between different populations, and finally, sensitivity analyses ensured the robustness of the findings. This study’s findings indicate the potential of the cMIND diet in deterring hypertension in elderly Chinese individuals, thereby justifying further extensive research to solidify this correlation and offer innovative methods to reduce the hypertension burden among the senior population.

## Limitations

Despite being the initial investigation into the association between the cMIND diet and hypertension among elderly Chinese individuals, the current study does possess certain limitations. Firstly, as a cross-sectional study, the causal relationship between the cMIND diet and hypertension could not be inferred. Secondly, the questionnaire was filled out based on self-report from the study participants, which may have biased information. Finally, hypertension is an intricate, multi-factorial chronic illness, with genetic predisposition serving as a significant intervening variable. Still, there was no information in the questionnaire related to the genetics of hypertension, which should be further included in future studies to explore the effect of genetic factors.

## Electronic supplementary material

Below is the link to the electronic supplementary material.

Supplementary Material 1

## Data Availability

The CLHLS data are available at https://opendata.pku.edu.cn/dataverse/CHADS.

## References

[1] Global report on (2023) Hypertension: the race against a silent killer. World Health Organ. :1–276

[2] Qu C, Liao S, Zhang J, Cao H, Zhang H, Zhang N et al (2024) 2024;10(2):143 – 53 Burden of cardiovascular disease among elderly: based on the global burden of disease study 2019. Eur Heart J Qual Care Clin Outcomes. http://www.ncbi.nlm.nih.gov/entrez/query.fcgi?cmd=Retrieve&db=pubmed&dopt=Abstract&list_uids=37296238&query_hl=110.1093/ehjqcco/qcad033 10.1093/ehjqcco/qcad033 PMC10904724 37296238

[3] Qiu Y, Ma J, Zhu J, Liu Y, Ren W, Zhang S et al (2023) 2023;10:1080682 Deaths and disability-adjusted life years of hypertension in china, south korea, and japan: a trend over the past 29 years. Front Cardiovasc Med. http://www.ncbi.nlm.nih.gov/entrez/query.fcgi?cmd=Retrieve&db=pubmed&dopt=Abstract&list_uids=37008311&query_hl=110.3389/fcvm.2023.1080682 10.3389/fcvm.2023.1080682 PMC10050598 37008311

[4] Zeng XZ, Jia N, Meng LB, Shi J, Li YY, Hu JB et al (2022) 2022;9:1057361 A study on the prevalence and related factors of frailty and pre-frailty in the older population with hypertension in china: a national cross-sectional study. Front Cardiovasc Med. http://www.ncbi.nlm.nih.gov/entrez/query.fcgi?cmd=Retrieve&db=pubmed&dopt=Abstract&list_uids=36712273&query_hl=110.3389/fcvm.2022.1057361 10.3389/fcvm.2022.1057361 PMC9877294 36712273

[5] Ma G, Luo A, Shen Z, Duan Y, Shi S, Zhong Z (2020) 2020;15(3):409 – 19 The status of medication literacy and associated factors of hypertensive patients in china: a cross-sectional study. Intern Emerg Med. http://www.ncbi.nlm.nih.gov/entrez/query.fcgi?cmd=Retrieve&db=pubmed&dopt=Abstract&list_uids=31650433&query_hl=110.1007/s11739-019-02187-0 10.1007/s11739-019-02187-0 PMC7165129 31650433

[6] Bernatova I, Liskova S, Bartekova M (2022) 2022;23(14) Hypertension and cardiovascular diseases: from etiopathogenesis to potential therapeutic targets. Int J Mol Sci. http://www.ncbi.nlm.nih.gov/entrez/query.fcgi?cmd=Retrieve&db=pubmed&dopt=Abstract&list_uids=35887089&query_hl=110.3390/ijms23147742 10.3390/ijms23147742 PMC9324881 35887089

[7] Shim JS, Oh K, Jung SJ, Kim HC (2020) Self-reported diet management and adherence to dietary guidelines in korean adults with hypertension. Korean Circ J. 2020. ;50(5):432 – 40. http://www.ncbi.nlm.nih.gov/entrez/query.fcgi?cmd=Retrieve&db=pubmed&dopt=Abstract&list_uids=32096363&query_hl=110.4070/kcj.2019.0230 10.4070/kcj.2019.0230 PMC7098818 32096363

[8] Zhou M, Wang H, Zeng X, Yin P, Zhu J, Chen W et al (2019) Mortality, morbidity, and risk factors in china and its provinces, 1990–2017: a systematic analysis for the global burden of disease study 2017. Lancet. 2019. ;394(10204):1145-58. http://www.ncbi.nlm.nih.gov/entrez/query.fcgi?cmd=Retrieve&db=pubmed&dopt=Abstract&list_uids=31248666&query_hl=110.1016/S0140-6736(19)30427-1 10.1016/S0140-6736(19)30427-1 PMC6891889 31248666

[9] Tozo T, Gisi ML, Brand C, Moreira C, Pereira BO, Leite N (2022) 2022;22(1):497 Family history of arterial hypertension and central adiposity: impact on blood pressure in schoolchildren. Bmc Pediatr. http://www.ncbi.nlm.nih.gov/entrez/query.fcgi?cmd=Retrieve&db=pubmed&dopt=Abstract&list_uids=35999624&query_hl=110.1186/s12887-022-03551-4 10.1186/s12887-022-03551-4 PMC9400321 35999624

[10] Ali N, Mahmud F, Akter SA, Islam S, Sumon AH, Barman DN et al (2022) 2022;24(10):1339-49 The prevalence of general obesity, abdominal obesity, and hypertension and its related risk factors among young adult students in bangladesh. J Clin Hypertens (Greenwich). http://www.ncbi.nlm.nih.gov/entrez/query.fcgi?cmd=Retrieve&db=pubmed&dopt=Abstract&list_uids=36000198&query_hl=110.1111/jch.14560 10.1111/jch.14560 PMC9581101 36000198

[11] Nakamura K, Barzi F, Lam TH, Huxley R, Feigin VL, Ueshima H et al (2008) Cigarette smoking, systolic blood pressure, and cardiovascular diseases in the asia-pacific region. Stroke. 2008. ;39(6):1694 – 702. http://www.ncbi.nlm.nih.gov/entrez/query.fcgi?cmd=Retrieve&db=pubmed&dopt=Abstract&list_uids=18323508&query_hl=110.1161/STROKEAHA.107.496752 10.1161/STROKEAHA.107.496752 18323508

[12] Nan X, Lu H, Wu J, Xue M, Qian Y, Wang W et al (2021) The interactive association between sodium intake, alcohol consumption and hypertension among elderly in northern china: a cross-sectional study. Bmc Geriatr. 2021. ;21(1):135. http://www.ncbi.nlm.nih.gov/entrez/query.fcgi?cmd=Retrieve&db=pubmed&dopt=Abstract&list_uids=33622268&query_hl=110.1186/s12877-021-02090-4 10.1186/s12877-021-02090-4 PMC7903677 33622268

[13] Wiener A, Rohr CS, Naor N, Villringer A, Okon-Singer H (2020) 2020;14:80 Emotion regulation in essential hypertension: roles of anxiety, stress, and the pulvinar. Front Behav Neurosci. http://www.ncbi.nlm.nih.gov/entrez/query.fcgi?cmd=Retrieve&db=pubmed&dopt=Abstract&list_uids=32547376&query_hl=110.3389/fnbeh.2020.00080 10.3389/fnbeh.2020.00080 PMC7270409 32547376

[14] Ncd countdown (2030) : worldwide trends in non-communicable disease mortality and progress towards sustainable development goal target 3.4. Lancet. 2018 2018;392(10152):1072-88. http://www.ncbi.nlm.nih.gov/entrez/query.fcgi?cmd=Retrieve&db=pubmed&dopt=Abstract&list_uids=30264707&query_hl=1 doi: 10.1016/S0140-6736(18)31992-5 10.1016/S0140-6736(18)31992-5 30264707

[15] De Pergola G, D’Alessandro A (2018) 2018;10(11) Influence of mediterranean diet on blood pressure. Nutrients. http://www.ncbi.nlm.nih.gov/entrez/query.fcgi?cmd=Retrieve&db=pubmed&dopt=Abstract&list_uids=30405063&query_hl=1 doi: 10.3390/nu10111700 10.3390/nu10111700 PMC6266047 30405063

[16] Onwuzo C, Olukorode JO, Omokore OA, Odunaike OS, Omiko R, Osaghae OW et al (2023) Dash diet: a review of its scientifically proven hypertension reduction and health benefits. Cureus. 2023. ;15(9):e44692. http://www.ncbi.nlm.nih.gov/entrez/query.fcgi?cmd=Retrieve&db=pubmed&dopt=Abstract&list_uids=37809159&query_hl=110.7759/cureus.44692 10.7759/cureus.44692 PMC10551663 37809159

[17] Hussain BM, Deierlein AL, Kanaya AM, Talegawkar SA, O’Connor JA, Gadgil MD et al (2023) 2023;15(16) Concordance between dash diet and hypertension: results from the mediators of atherosclerosis in south asians living in america (masala) study. Nutrients. http://www.ncbi.nlm.nih.gov/entrez/query.fcgi?cmd=Retrieve&db=pubmed&dopt=Abstract&list_uids=37630801&query_hl=110.3390/nu15163611 10.3390/nu15163611 PMC10458588 37630801

[18] Francisco SC, Araújo LF, Griep RH, Chor D, Molina M, Mil JG et al (2020) 2020;123(9):1068-77 Adherence to the dietary approaches to stop hypertension (dash) and hypertension risk: results of the longitudinal study of adult health (elsa-brasil). Br J Nutr. http://www.ncbi.nlm.nih.gov/entrez/query.fcgi?cmd=Retrieve&db=pubmed&dopt=Abstract&list_uids=31959262&query_hl=110.1017/S0007114520000124 10.1017/S0007114520000124 31959262

[19] Lăcătușu CM, Grigorescu ED, Floria M, Onofriescu A, Mihai BM (2019) 2019;16(6) The mediterranean diet: from an environment-driven food culture to an emerging medical prescription. Int J Environ Res Public Health. http://www.ncbi.nlm.nih.gov/entrez/query.fcgi?cmd=Retrieve&db=pubmed&dopt=Abstract&list_uids=30875998&query_hl=110.3390/ijerph16060942 10.3390/ijerph16060942 PMC6466433 30875998

[20] Altawili AA, Altawili M, Alwadai AM, Alahmadi AS, Alshehri A, Muyini BH et al (2023) An exploration of dietary strategies for hypertension management: a narrative review. Cureus. 2023. ;15(12):e50130. http://www.ncbi.nlm.nih.gov/entrez/query.fcgi?cmd=Retrieve&db=pubmed&dopt=Abstract&list_uids=38186513&query_hl=110.7759/cureus.50130 10.7759/cureus.50130 PMC10771610 38186513

[21] Richardson LA, Izuora K, Basu A (2022) 2022;19(19) Mediterranean diet and its association with cardiovascular disease risk factors: a scoping review. Int J Environ Res Public Health. http://www.ncbi.nlm.nih.gov/entrez/query.fcgi?cmd=Retrieve&db=pubmed&dopt=Abstract&list_uids=36232062&query_hl=110.3390/ijerph191912762 10.3390/ijerph191912762 PMC9566634 36232062

[22] Martinez-Lacoba R, Pardo-Garcia I, Amo-Saus E, Escribano-Sotos F (2018) Mediterranean diet and health outcomes: a systematic meta-review. Eur J Public Health. 2018. ;28(5):955 – 61. http://www.ncbi.nlm.nih.gov/entrez/query.fcgi?cmd=Retrieve&db=pubmed&dopt=Abstract&list_uids=29992229&query_hl=110.1093/eurpub/cky113 10.1093/eurpub/cky113 29992229

[23] Filippou CD, Tsioufis CP, Thomopoulos CG, Mihas CC, Dimitriadis KS, Sotiropoulou LI et al (2020) Dietary approaches to stop hypertension (dash) diet and blood pressure reduction in adults with and without hypertension: a systematic review and meta-analysis of randomized controlled trials. Adv Nutr. 2020. ;11(5):1150-60. http://www.ncbi.nlm.nih.gov/entrez/query.fcgi?cmd=Retrieve&db=pubmed&dopt=Abstract&list_uids=32330233&query_hl=110.1093/advances/nmaa041 10.1093/advances/nmaa041 PMC7490167 32330233

[24] Motamedi A, Ekramzadeh M, Bahramali E, Farjam M, Homayounfar R (2021) Diet quality in relation to the risk of hypertension among iranian adults: cross-sectional analysis of fasa persian cohort study. Nutr J. 2021. ;20(1):57. http://www.ncbi.nlm.nih.gov/entrez/query.fcgi?cmd=Retrieve&db=pubmed&dopt=Abstract&list_uids=34174902&query_hl=110.1186/s12937-021-00717-1 10.1186/s12937-021-00717-1 PMC8236133 34174902

[25] Magriplis E, Panagiotakos D, Kyrou I, Tsioufis C, Mitsopoulou AV, Karageorgou D et al (2020) 2020;12(3) Presence of hypertension is reduced by mediterranean diet adherence in all individuals with a more pronounced effect in the obese: the hellenic national nutrition and health survey (hnnhs). Nutrients. http://www.ncbi.nlm.nih.gov/entrez/query.fcgi?cmd=Retrieve&db=pubmed&dopt=Abstract&list_uids=32209978&query_hl=110.3390/nu12030853 10.3390/nu12030853 PMC7146360 32209978

[26] Zhang Y, Wang Y, Chen Y, Zhou J, Xu L, Xu K et al (2021) 2021;18(23) Associations of dietary patterns and risk of hypertension in southwest china: a prospective cohort study. Int J Environ Res Public Health. http://www.ncbi.nlm.nih.gov/entrez/query.fcgi?cmd=Retrieve&db=pubmed&dopt=Abstract&list_uids=34886102&query_hl=110.3390/ijerph182312378 10.3390/ijerph182312378 PMC8656527 34886102

[27] Wu Y, Xie Y, Yuan Y, Xiong R, Hu Y, Ning K et al (2023) The mediterranean diet and age-related eye diseases: a systematic review. Nutrients. 2023. ;15(9). http://www.ncbi.nlm.nih.gov/entrez/query.fcgi?cmd=Retrieve&db=pubmed&dopt=Abstract&list_uids=37432187&query_hl=110.3390/nu15092043 10.3390/nu15092043 PMC10181476 37432187

[28] Jia L, Lu H, Wu J, Wang X, Wang W, Du M et al (2020) 2020;20(1):1165 Association between diet quality and obesity indicators among the working-age adults in inner mongolia, northern china: a cross-sectional study. Bmc Public Health. http://www.ncbi.nlm.nih.gov/entrez/query.fcgi?cmd=Retrieve&db=pubmed&dopt=Abstract&list_uids=32711506&query_hl=110.1186/s12889-020-09281-5 10.1186/s12889-020-09281-5 PMC7382798 32711506

[29] Huang X, Aihemaitijiang S, Ye C, Halimulati M, Wang R, Zhang Z (2022) 2022;26(8):760 – 70 Development of the cmind diet and its association with cognitive impairment in older chinese people. J Nutr Health Aging. http://www.ncbi.nlm.nih.gov/entrez/query.fcgi?cmd=Retrieve&db=pubmed&dopt=Abstract&list_uids=35934820&query_hl=110.1007/s12603-022-1829-110.1007/s12603-022-1829-135934820

[30] Wang R, Ye C, Huang X, Halimulati M, Sun M, Ma Y et al (2023) Cmind diet, indoor air pollution, and depression: a cohort study based on the clhls from 2011 to 2018. Nutrients. 2023. ;15(5). http://www.ncbi.nlm.nih.gov/entrez/query.fcgi?cmd=Retrieve&db=pubmed&dopt=Abstract&list_uids=36904202&query_hl=110.3390/nu15051203 10.3390/nu15051203 PMC10005708 36904202

[31] Lin W, Zhou X, Liu X (2024) Association of adherence to the chinese version of the mind diet with reduced cognitive decline in older chinese individuals: analysis of the chinese longitudinal healthy longevity survey. J Nutr Health Aging. 2024. ;28(2):100024. http://www.ncbi.nlm.nih.gov/entrez/query.fcgi?cmd=Retrieve&db=pubmed&dopt=Abstract&list_uids=38388105&query_hl=110.1016/j.jnha.2023.10002410.1016/j.jnha.2023.10002438388105

[32] Wang J, Lin X, Bloomgarden ZT, Ning G (2020) 2020;12(5):365 – 71 The jiangnan diet, a healthy diet pattern for chinese. J Diabetes. http://www.ncbi.nlm.nih.gov/entrez/query.fcgi?cmd=Retrieve&db=pubmed&dopt=Abstract&list_uids=31846221&query_hl=110.1111/1753-0407.13015 10.1111/1753-0407.13015 PMC7216939 31846221

[33] Li J, Ding H, Wang Z, El-Ansary D, Adams R, Han J et al (2022) 2022;9:831109 Translation, cultural adaptation, reliability, and validity testing of a chinese version of the self-administered mediterranean diet scale. Front Nutr. http://www.ncbi.nlm.nih.gov/entrez/query.fcgi?cmd=Retrieve&db=pubmed&dopt=Abstract&list_uids=35419397&query_hl=110.3389/fnut.2022.831109 10.3389/fnut.2022.831109 PMC8996054 35419397

[34] Song Y, Chang Z, Cui K, Song C, Cai Z, Shi B et al (2023) 2023;10:1129667 The value of the mind diet in the primary and secondary prevention of hypertension: a cross-sectional and longitudinal cohort study from nhanes analysis. Front Nutr. http://www.ncbi.nlm.nih.gov/entrez/query.fcgi?cmd=Retrieve&db=pubmed&dopt=Abstract&list_uids=36998902&query_hl=110.3389/fnut.2023.1129667 10.3389/fnut.2023.1129667 PMC10043250 36998902

[35] Deng Y, Gao Q, Yang D, Hua H, Wang N, Ou F et al (2020) Association between biomass fuel use and risk of hypertension among chinese older people: a cohort study. Environ Int. 2020. ;138:105620. http://www.ncbi.nlm.nih.gov/entrez/query.fcgi?cmd=Retrieve&db=pubmed&dopt=Abstract&list_uids=32179315&query_hl=110.1016/j.envint.2020.105620 10.1016/j.envint.2020.105620 32179315

[36] Yang F, Fu M, Hu Q, Guo J (2023) 2023;14:1081209 The associations between cognitive function and depressive symptoms among older chinese population: a cohort study. Front Psychiatry. http://www.ncbi.nlm.nih.gov/entrez/query.fcgi?cmd=Retrieve&db=pubmed&dopt=Abstract&list_uids=37091713&query_hl=110.3389/fpsyt.2023.1081209 10.3389/fpsyt.2023.1081209 PMC10117645 37091713

[37] Tao Z, Feng Y, Liu J, Tao L (2023) Trends and disparities in sleep quality and duration in older adults in china from 2008 to 2018: a national observational study. Front Public Health. 2023. ;11:998699. http://www.ncbi.nlm.nih.gov/entrez/query.fcgi?cmd=Retrieve&db=pubmed&dopt=Abstract&list_uids=36875376&query_hl=110.3389/fpubh.2023.998699 10.3389/fpubh.2023.998699 PMC9982158 36875376

[38] Zeng Y, Feng Q, Hesketh T, Christensen K, Vaupel JW (2017) 2017;389(10079):1619-29 Survival, disabilities in activities of daily living, and physical and cognitive functioning among the oldest-old in china: a cohort study. Lancet. http://www.ncbi.nlm.nih.gov/entrez/query.fcgi?cmd=Retrieve&db=pubmed&dopt=Abstract&list_uids=28285816&query_hl=110.1016/S0140-6736(17)30548-2 10.1016/S0140-6736(17)30548-2 PMC5406246 28285816

[39] Eckel RH, Jakicic JM, Ard JD, de Jesus JM, Houston MN, Hubbard VS et al (2014) 2013 aha/acc guideline on lifestyle management to reduce cardiovascular risk: a report of the american college of cardiology/american heart association task force on practice guidelines. Circulation. 2014. ;129(25 Suppl 2):S76-99. http://www.ncbi.nlm.nih.gov/entrez/query.fcgi?cmd=Retrieve&db=pubmed&dopt=Abstract&list_uids=24222015&query_hl=110.1161/01.cir.0000437740.48606.d1 10.1161/01.cir.0000437740.48606.d1 24222015

[40] Liu SS, Kim JY, Park JH, Kim S, Lee K, Bae WK et al (2021) 2021;42(5):382 – 89 Fruit intake and changes of cardio-metabolic risk factors in people with obesity. Korean J Fam Med. http://www.ncbi.nlm.nih.gov/entrez/query.fcgi?cmd=Retrieve&db=pubmed&dopt=Abstract&list_uids=34607414&query_hl=110.4082/kjfm.20.0205 10.4082/kjfm.20.0205 PMC8490179 34607414

[41] Liu X, Lai H, Mi B, Qi X, Gan W, Du H (2020) Associations of coarse grain intake with undiagnosed hypertension among chinese adults: results from the china kadoorie biobank. Nutrients. 2020. ;12(12). http://www.ncbi.nlm.nih.gov/entrez/query.fcgi?cmd=Retrieve&db=pubmed&dopt=Abstract&list_uids=33322167&query_hl=110.3390/nu12123814 10.3390/nu12123814 PMC7764616 33322167

[42] Zhao Y, Tang C, Tang W, Zhang X, Jiang X, Duoji Z et al (2023) The association between tea consumption and blood pressure in the adult population in southwest china. Bmc Public Health. 2023. ;23(1):476. http://www.ncbi.nlm.nih.gov/entrez/query.fcgi?cmd=Retrieve&db=pubmed&dopt=Abstract&list_uids=36915113&query_hl=110.1186/s12889-023-15315-5 10.1186/s12889-023-15315-5 PMC10010002 36915113

[43] Lelong H, Blacher J, Baudry J, Adriouch S, Galan P, Fezeu L et al (2017) Individual and combined effects of dietary factors on risk of incident hypertension: prospective analysis from the nutrinet-santé cohort. Hypertension. 2017. ;70(4):712 – 20. http://www.ncbi.nlm.nih.gov/entrez/query.fcgi?cmd=Retrieve&db=pubmed&dopt=Abstract&list_uids=28760943&query_hl=110.1161/HYPERTENSIONAHA.117.09622 10.1161/HYPERTENSIONAHA.117.09622 28760943

[44] PAGE IH. Pathogenesis of arterial hypertension. J Am Med Assoc (1949) 1949;140(5):451 – 58 http://www.ncbi.nlm.nih.gov/entrez/query.fcgi?cmd=Retrieve&db=pubmed&dopt=Abstract&list_uids=18129851&query_hl=110.1001/jama.1949.02900400005002 10.1001/jama.1949.02900400005002 18129851

[45] Wang X, Khalil RA (2018) 2018;81:241–330 Matrix metalloproteinases, vascular remodeling, and vascular disease. Adv Pharmacol. http://www.ncbi.nlm.nih.gov/entrez/query.fcgi?cmd=Retrieve&db=pubmed&dopt=Abstract&list_uids=29310800&query_hl=110.1016/bs.apha.2017.08.002 10.1016/bs.apha.2017.08.002 PMC5765875 29310800

[46] Stewart R (2018) 2018;379(14):1388 Primary prevention of cardiovascular disease with a mediterranean diet supplemented with extra-virgin olive oil or nuts. N Engl J Med. http://www.ncbi.nlm.nih.gov/entrez/query.fcgi?cmd=Retrieve&db=pubmed&dopt=Abstract&list_uids=30285333&query_hl=110.1056/NEJMc1809971 10.1056/NEJMc1809971 30285333

[47] González-Rámila S, Sarriá B, Seguido MA, García-Cordero J, Mateos R, Bravo L (2023) 2023;62(2):589–603 Olive pomace oil can improve blood lipid profile: a randomized, blind, crossover, controlled clinical trial in healthy and at-risk volunteers. Eur J Nutr. http://www.ncbi.nlm.nih.gov/entrez/query.fcgi?cmd=Retrieve&db=pubmed&dopt=Abstract&list_uids=36153442&query_hl=110.1007/s00394-022-03001-y 10.1007/s00394-022-03001-y PMC9941261 36153442

[48] Aiello P, Peluso I, Di Giacomo S, Di Sotto A, Villaño VD (2022) Body composition and metabolic status of italian and spanish university students: relationship with fruit and vegetable consumption. Nutrients. 2022. ;14(16). http://www.ncbi.nlm.nih.gov/entrez/query.fcgi?cmd=Retrieve&db=pubmed&dopt=Abstract&list_uids=36014802&query_hl=110.3390/nu14163296 10.3390/nu14163296 PMC9415832 36014802

[49] Tapsell L, Sabaté J, Martínez R, Llavanera M, Neale E, Salas-Huetos A (2023) Novel lines of research on the environmental and human health impacts of nut consumption. Nutrients. 2023. ;15(4). http://www.ncbi.nlm.nih.gov/entrez/query.fcgi?cmd=Retrieve&db=pubmed&dopt=Abstract&list_uids=36839312&query_hl=110.3390/nu15040955 10.3390/nu15040955 PMC9964796 36839312

[50] Wang DD, Leung CW, Li Y, Ding EL, Chiuve SE, Hu FB et al (2014) 2014;174(10):1587-95 Trends in dietary quality among adults in the united states, 1999 through 2010. Jama Intern Med. http://www.ncbi.nlm.nih.gov/entrez/query.fcgi?cmd=Retrieve&db=pubmed&dopt=Abstract&list_uids=25179639&query_hl=110.1001/jamainternmed.2014.3422 10.1001/jamainternmed.2014.3422 PMC5924699 25179639

[51] Imamura M, Sasaki H, Shinto T, Tahara Y, Makino S, Kuwahara M et al (2022) 2022;9:853118 Association between na, k, and lipid intake in each meal and blood pressure. Front Nutr. http://www.ncbi.nlm.nih.gov/entrez/query.fcgi?cmd=Retrieve&db=pubmed&dopt=Abstract&list_uids=35308273&query_hl=110.3389/fnut.2022.853118 10.3389/fnut.2022.853118 PMC8931534 35308273

[52] Mannon EC, Muller PR, Sun J, Bush WB, Coleman A, Ocasio H et al (2024) 2024;138(4):189–203 Nahco3 loading causes increased arterial pressure and kidney damage in rats with chronic kidney disease. Clin Sci (Lond). http://www.ncbi.nlm.nih.gov/entrez/query.fcgi?cmd=Retrieve&db=pubmed&dopt=Abstract&list_uids=38300615&query_hl=110.1042/CS20231709 10.1042/CS20231709 38300615

[53] Hall JE, Do CJ, Da SA, Wang Z, Hall ME (2019) 2019;15(6):367 – 85 Obesity, kidney dysfunction and hypertension: mechanistic links. Nat Rev Nephrol. http://www.ncbi.nlm.nih.gov/entrez/query.fcgi?cmd=Retrieve&db=pubmed&dopt=Abstract&list_uids=31015582&query_hl=110.1038/s41581-019-0145-4 10.1038/s41581-019-0145-4 PMC7278043 31015582

[54] Gonzalez-Vicente A, Hong N, Garvin JL (2019) 2019;317(2):F444-55 Effects of reactive oxygen species on renal tubular transport. Am J Physiol Renal Physiol. http://www.ncbi.nlm.nih.gov/entrez/query.fcgi?cmd=Retrieve&db=pubmed&dopt=Abstract&list_uids=31215804&query_hl=110.1152/ajprenal.00604.2018 10.1152/ajprenal.00604.2018 PMC6732451 31215804

[55] Loperena R, Harrison DG (2017) 2017;101(1):169 – 93 Oxidative stress and hypertensive diseases. Med Clin North Am. http://www.ncbi.nlm.nih.gov/entrez/query.fcgi?cmd=Retrieve&db=pubmed&dopt=Abstract&list_uids=27884227&query_hl=110.1016/j.mcna.2016.08.004 10.1016/j.mcna.2016.08.004 PMC5125524 27884227

[56] George S, Abrahamse H (2020) 2020;9(11) Redox potential of antioxidants in cancer progression and prevention. Antioxidants (Basel). http://www.ncbi.nlm.nih.gov/entrez/query.fcgi?cmd=Retrieve&db=pubmed&dopt=Abstract&list_uids=33233630&query_hl=110.3390/antiox9111156 10.3390/antiox9111156 PMC7699713 33233630

[57] Touyz RM, Rios FJ, Alves-Lopes R, Neves KB, Camargo LL, Montezano AC (2020) 2020;36(5):659 – 70 Oxidative stress: a unifying paradigm in hypertension. Can J Cardiol. http://www.ncbi.nlm.nih.gov/entrez/query.fcgi?cmd=Retrieve&db=pubmed&dopt=Abstract&list_uids=32389339&query_hl=110.1016/j.cjca.2020.02.081 10.1016/j.cjca.2020.02.081 PMC7225748 32389339

[58] Yu TF, Hou ZH, Wang HL, Chang SY, Song XY, Zheng WJ et al Soybean steroids improve crop abiotic stress tolerance and increase yield. Plant Biotechnol J. 2024 2024 Apr 10. http://www.ncbi.nlm.nih.gov/entrez/query.fcgi?cmd=Retrieve&db=pubmed&dopt=Abstract&list_uids=38600703&query_hl=110.1111/pbi.14349 10.1111/pbi.14349 PMC11258977 38600703

[59] Varghese JS, Ghosh A, Stein A, Narayan KV, Patel S (2024) The association of hypertension among married indian couples: a nationally representative cross-sectional study. Res Sq. 2024 Feb 2. http://www.ncbi.nlm.nih.gov/entrez/query.fcgi?cmd=Retrieve&db=pubmed&dopt=Abstract&list_uids=38352475&query_hl=110.21203/rs.3.rs-3865512/v1 10.1038/s41598-024-61169-1 PMC11074266 38710852

[60] Adeke AS, Chori BS, Neupane D, Sharman JE, Odili AN (2024) 2024;38(4):365 – 70 Socio-demographic and lifestyle factors associated with hypertension in nigeria: results from a country-wide survey. J Hum Hypertens. http://www.ncbi.nlm.nih.gov/entrez/query.fcgi?cmd=Retrieve&db=pubmed&dopt=Abstract&list_uids=35332218&query_hl=110.1038/s41371-022-00673-1 10.1038/s41371-022-00673-1 PMC11001570 35332218

[61] Shiffman D, Louie JZ, Devlin JJ, Rowland CM, Mora S (2020) Concordance of cardiovascular risk factors and behaviors in a multiethnic us nationwide cohort of married couples and domestic partners. JAMA Netw Open. 2020. ;3(10):e2022119. http://www.ncbi.nlm.nih.gov/entrez/query.fcgi?cmd=Retrieve&db=pubmed&dopt=Abstract&list_uids=33104207&query_hl=110.1001/jamanetworkopen.2020.22119 10.1001/jamanetworkopen.2020.22119 PMC7588939 33104207

[62] Vallée A (2023) 2023;21:61 Associations between smoking and alcohol consumption with blood pressure in a middle-aged population. Tob Induc Dis. http://www.ncbi.nlm.nih.gov/entrez/query.fcgi?cmd=Retrieve&db=pubmed&dopt=Abstract&list_uids=37215190&query_hl=110.18332/tid/162440 10.18332/tid/162440 PMC10193384 37215190

[63] Nagao T, Nogawa K, Sakata K, Morimoto H, Morita K, Watanabe Y et al (2021) 2021;18(22) Effects of alcohol consumption and smoking on the onset of hypertension in a long-term longitudinal study in a male workers’ cohort. Int J Environ Res Public Health. http://www.ncbi.nlm.nih.gov/entrez/query.fcgi?cmd=Retrieve&db=pubmed&dopt=Abstract&list_uids=34831535&query_hl=110.3390/ijerph182211781 10.3390/ijerph182211781 PMC8619602 34831535

[64] Bakris G, Sorrentino M (2018) 2018;378(6):497 – 99 Redefining hypertension - assessing the new blood-pressure guidelines. N Engl J Med. http://www.ncbi.nlm.nih.gov/entrez/query.fcgi?cmd=Retrieve&db=pubmed&dopt=Abstract&list_uids=29341841&query_hl=1 doi: 10.1056/NEJMp1716193 10.1056/NEJMp1716193 29341841

[65] Devranis P, Vassilopoulou Ε, Tsironis V, Sotiriadis PM, Chourdakis M, Aivaliotis M et al (2023) 2023;13(1) Mediterranean diet, ketogenic diet or mind diet for aging populations with cognitive decline: a systematic review. Life (Basel). http://www.ncbi.nlm.nih.gov/entrez/query.fcgi?cmd=Retrieve&db=pubmed&dopt=Abstract&list_uids=36676122&query_hl=110.3390/life13010173 10.3390/life13010173 PMC9866105 36676122

[66] Boumenna T, Scott TM, Lee JS, Zhang X, Kriebel D, Tucker KL et al (2022) 2022;77(3):605 – 13 Mind diet and cognitive function in puerto rican older adults. J Gerontol A Biol Sci Med Sci. http://www.ncbi.nlm.nih.gov/entrez/query.fcgi?cmd=Retrieve&db=pubmed&dopt=Abstract&list_uids=34551094&query_hl=110.1093/gerona/glab261 10.1093/gerona/glab261 PMC8893189 34551094

[67] Bosworth HB, Olsen MK, Grubber JM, Neary AM, Orr MM, Powers BJ et al (2009) 2009;151(10):687 – 95 Two self-management interventions to improve hypertension control: a randomized trial. Ann Intern Med. http://www.ncbi.nlm.nih.gov/entrez/query.fcgi?cmd=Retrieve&db=pubmed&dopt=Abstract&list_uids=19920269&query_hl=110.7326/0003-4819-151-10-200911170-00148 10.1059/0003-4819-151-10-200911170-00148 PMC2892337 19920269

[68] de Ridder D, Kroese F, Evers C, Adriaanse M, Gillebaart M (2017) Healthy diet: health impact, prevalence, correlates, and interventions. Psychol Health. 2017. ;32(8):907 – 41. http://www.ncbi.nlm.nih.gov/entrez/query.fcgi?cmd=Retrieve&db=pubmed&dopt=Abstract&list_uids=28447854&query_hl=110.1080/08870446.2017.1316849 10.1080/08870446.2017.1316849 28447854

