# Supplementary material for: Association between cMIND diet and hypertension among older adults in China: a nationwide survey
Source: Aging Clin Exp Res. 2024 Sep 5;36(1):182. doi: 10.1007/s40520-024-02842-3 (PMC11377468; doi:10.1007/s40520-024-02842-3)
Supplement: Supplementary file 1 — Supplementary Material 1 [file 40520_2024_2842_MOESM1_ESM.docx]

**Supplementary Information**

**Table S1.**  Measurement of covariates

| Variables | Questionnaire | Assigned the values |
| --- | --- | --- |
| Sex | What is your sex? | 0=male; 1=female |
| Age | How old are you? | 0=65-79; 1=≥80 |
| Residence | What is your current area of residence? | 0= urban; 1= rural |
| Living arrangements | What is your current living arrangement? | 1=with household member(s); 2=alone; 3=in an institution |
| Education | How many years of schooling did you have? | 0=0; 1=1-6; 2=≥7 |
| Economic status | How do you rate your economic status compared with other local people? | 0=not wealthy; 1=general; 2=wealthy |
| Marital status | What is your current marital status? | 0=unmarried; 1=married |
| Smoking | Are you smoking now? | 0=no; 1=yes |
| Drinking | Are you drinking now? | 0=no; 1=yes |
| Exercise | Do you exercise now? | 0=no; 1=yes |
| Body-mass index(BMI) | Weights(kg)/Height (m)2 | 0=＜18.5; 1=18.5-23.9; 2=>23.9 |
| Diabetes | Do you have diabetes? | 0=no; 1=yes |
| Cardiovascular disease | Do you have stroke or cardiovascular diseases? | 0=no; 1=yes |
| Cognitive impairment | Do you have cognitive impairment? | 0=no; 1=yes |

**Table S2.**  Sensitivity analysis of cMIND diet and hypertension

| Variables | OR（95%CI） | P |
| --- | --- | --- |
| The elderly people with diabetes and heart disease were excluded |  |  |
| cMIND diet was used as a continuous variable | 0.962（0.928，0.998） | 0.040 |
| cMIND diet was used as a categorical variable ( VS. Low ) |  |  |
| Medium | 0.898（0.794，1.017） | 0.090 |
| High | 0.835（0.724，0.964） | 0.014 |
| The elderly with cognitive impairment were further excluded |  |  |
| cMIND diet was used as a continuous variable | 0.953（0.913，0.995） | 0.029 |
| cMIND diet was used as a categorical variable ( VS. Low ) |  |  |
| Medium | 0.902（0.776，1.050） | 0.183 |
| High | 0.809（0.684，0.957） | 0.014 |
